# Comparison of VATS and Thoracotomy Results in Mediastinal Neurogenic Tumors

**DOI:** 10.5152/eurasianjmed.2021.20357

**Published:** 2021-10

**Authors:** Ali Bilal Ulas, Yener Aydin, Atilla Eroglu, Betul Gundogdu

**Affiliations:** 1 Department of Thoracic Surgery, Atatürk University School of Medicine, Erzurum, Turkey; 2 Department of Pathology, Atatürk University School of Medicine, Erzurum, Turkey

**Keywords:** Mediastinum, neurogenic tumors, thoracotomy, thoracoscopy

## Abstract

**Objective:**

In this study, we aimed to compare the results of patients who underwent surgery by thoracotomy and Video-assisted thoracoscopic surgery (VATS) in mediastinal neurogenic tumors.

**Materials and Methods:**

Twenty-six consecutive cases (12 males and 14 females; mean age 39.4 ± 22.3 years; range 1–72 years) who were histopathologically diagnosed as having mediastinal neurogenic tumors between January 2000 and August 2020 were included in a single-center, retrospective study.

**Results:**

There were 5 (19.2%) children and 21 (80.8%) adults. Lesions in all cases were located in the posterior mediastinum. Schwannoma was detected histopathologically in 18 cases (69.2%), and all of these cases were adult patients. Resection was performed by thoracotomy in 14 cases (7 right and 7 left) and 12 cases by thoracoscopy (7 right and 5 left). The mean tumor size was 7.4 ± 1.9 cm (range 5–12 cm) in the thoracotomy group and 4.3 ± 1.9 cm (range 2–7 cm) in the VATS group (*P* = .001). Mean operative time was 101.7 ± 27.8 min (range 70–150 min) in the thoracotomy group and 77.9 ± 24.3 min (range 60–150 min) in the VATS group (*P* = .014). Mean postoperative hospital stay was 7.4 ± 4.0 days (range 3–20 days) in the thoracotomy group and 4.7 ± 1.7 days (range 2–7 days) in the VATS group (*P* = .040).

**Conclusion:**

Most of the mediastinal neurogenic tumors are benign and surgical resection is required in their treatment. With increasing experience, resection can be performed thoracoscopically in most cases.

## Introduction

Neurogenic tumors develop in tissues that originate from the embryonic neural crest. They are usually located in the upper mediastinum, especially in the posterior mediastinum and paravertebral sulcus. They are linked to the sympathetic chain, spinal nerve, or intercostal nerves but can be seen wherever nerve tissue is located, including the intraparenchymal, endotracheal, and chest wall. While the majority of neurogenic tumors in adults originate from nerve sheath, those with autonomic ganglia origin are more common in children.^1,2^ Neurogenic tumors constitute 12–24% of all primary mediastinal tumors.^3^ Most of the cases in adults are benign forms. While the malignant form accounts for 6% of the cases in adults, it constitutes 40–60% of the cases in children up to 5 years of age.^3,4^

Although the main treatment for neurogenic tumors of the mediastinum is complete resection of the tumor, sometimes multimodal treatment may be required. In this study, we aimed to compare the results of cases operated by thoracotomy and VATS in mediastinal neurogenic tumors.

## Materials and Methods

In a single-center, retrospective study, 26 consecutive cases (12 males and 14 females; mean age 39.4 ± 22.3 years; range 1–72 years) who were operated in Ataturk University Faculty of Medicine Thoracic Surgery Clinic between January 2000 and August 2020 and diagnosed histopathologically with mediastinal neurogenic tumor were included. Medical histories of all cases were taken, and physical examination findings were recorded. Complete blood count, biochemical parameters, and coagulation tests were performed in all cases. Radiologically, all cases were evaluated with posteroanterior chest radiography and computed tomography (CT) findings. Also, 9 cases were evaluated by MRI, 5 cases by PET-CT, and 2 cases by spinal angiography. Data regarding the age, gender, symptoms, tumor localization, diagnostic method, radiological findings, treatments applied, and survival results of the patients were recorded.

Written informed consent was obtained from each patient. The study protocol was approved by the Atatürk University Faculty of Medicine Ethics Committee (B.30.2.ATA.0.01.00/429). The study was conducted under the principles of the Declaration of Helsinki.

## Statistical Analysis

The IBM SPSS version 20.0 software (IBM SPSS Corp.; Armonk, NY, USA) was used for statistical analyses. Data are presented as mean, standard deviation, median and with the minimum and maximum values, numbers, and percentages. A *P* value < .05 was considered as statistically significant.

## Results

There were 5 (19.2%) children and 21 (80.8%) adults. The most common symptoms were back pain (8 cases, 30.8%) and chest pain (6 cases, 23.1). While 3 patients presented with complaints of cough, 2 patients with fever, 2 patients with weakness and anorexia, and 1 patient with hiccups, 9 patients (34.6%) were asymptomatic in whom neurogenic tumors were detected incidentally. Lesions were located in the posterior mediastinum in all cases.

**Figure 1.**
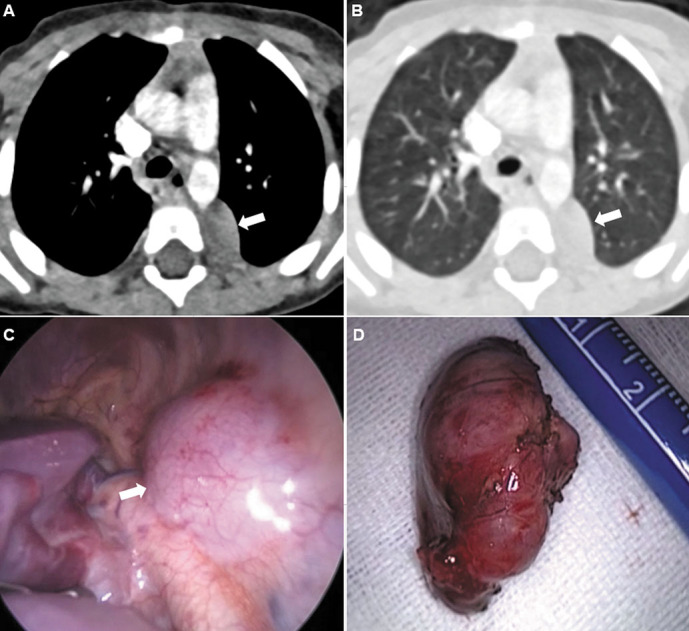
A 2-year-old male patient has a soft tissue density of approximately 23 × 13 mm in the posterior mediastinum in the left posterolateral part of the aorta on thoracic CT (A, B). The lesion was thoracoscopically resected (B, C). Ganglioneuroblastoma was detected in the histopathological examination of the mass sample

**Figure 2.**
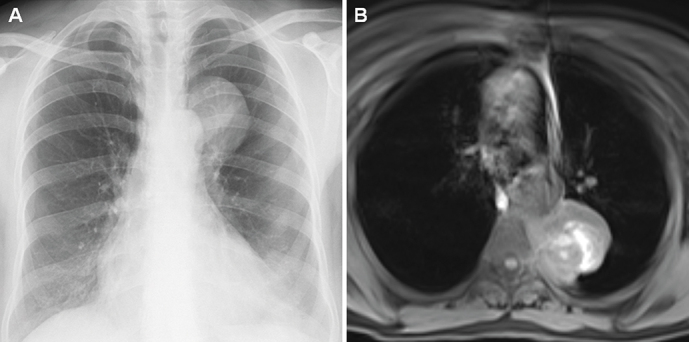
A well-circumscribed lesion on the left is seen on posteroanterior chest radiography in a 59-year-old female patient (A). MRI showed a mass lesion with cystic necrotic hemorrhagic changes with an axial diameter of 43 × 51 mm located in the left paravertebral area adjacent to the aortic isthmus and thoracic aorta (B). Paraganglioma was found in the histopathological examination of the case

**Figure 3.**
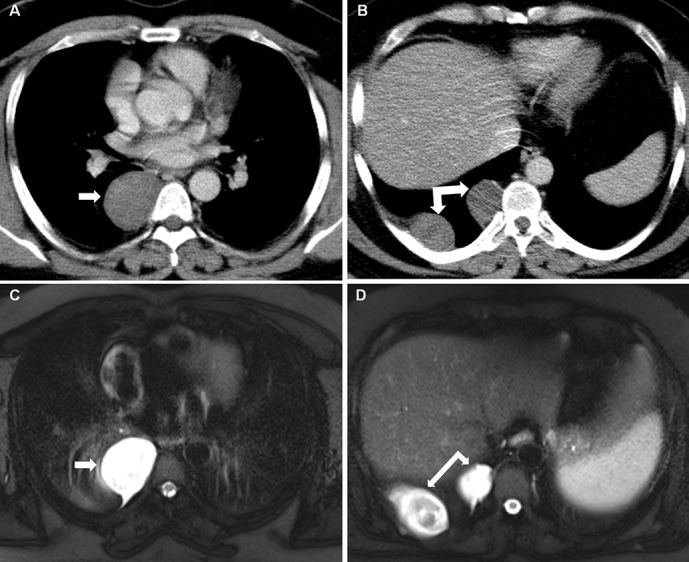
In a 42-year-old male case, hypodense mass appearances with three soft tissue densities, the largest of which is 77 × 39 mm in size with a pleural base, extra-axial located on the right, are observed on thoracic CT (A, B). The lesion causes destruction and invasion in the adjacent rib. In MRI, it was determined that the lesion caused partially restricted diffusion and increased heterogeneous contrast enhancement on postcontrast sections (C, D). Neurofibroma was detected in the histopathological examination of the case

Schwannoma was detected histopathologically in 18 cases (69.2%), and all of these cases were adult patients. Ganglioneuromas were detected in 3 cases (ages 3, 6, and 7 years). Ganglioneurobalastoma was detected in 2 cases (2 and 42 years old), paraganglioma in 1 (59 years old), neurofibroma in 1 (42 years old), and neuroblastoma in 1 (1-year-old). While the tumor was located on the right side in 14 of the cases, it was localized in the left hemithorax in 12 cases. In one case, hemothorax accompanied the tumor (Figures 1–3).

Resection was performed by thoracotomy in 14 of the cases (7 right and 7 left) and in 12 patients by thoracoscopy (7 right and 5 left). While the mediastinal tumor was resected in all cases, additional rib resection was performed in 2 cases, and corpectomy and fusion in 1 case. One of the patients who underwent VATS (paraganglioma) was converted to thoracotomy due to adhesions. The mean tumor size was 7.4 ± 1.9 cm (range 5–12 cm) in the thoracotomy group and 4.3 ± 1.9 cm (range 2–7 cm) in the VATS group (*P* = .001). Mean operative time was 101.7 ± 27.8 min (range 70–150 min) in the thoracotomy group and 77.9 ± 24.3 min (range 60–150 min) in the VATS group (*P* = .014). Mean postoperative hospital stay was 7.4 ± 4.0 days (range 3–20 days) in the thoracotomy group and 4.7 ± 1.7 days (range 2–7 days) in the VATS group (*P* = .040).

No complications or mortality were observed in the early period. Recurrence was observed 1 year postoperatively in 2 cases with ganglioneuroblastoma. In follow-up, 2 cases with recurrence of ganglioneuroblastoma and 1 case with paraganglioma (due to a secondary brain neuroendocrine tumor) have died (Table 1).

## Discussion

Neurogenic tumors are usually benign, slow-growing tumors that usually occur in the intercostal or sympathetic nerve course and are located in the posterior mediastinum in 90–95% of cases.^3^ In our study, all of the tumors were localized in the posterior mediastinum and the mean age of 26 patients was 39.4 years (range 1–72). Chen et al.^5^ reported the mean age as 47.0 years in their study of 121 cases. Takeda et al.^6^ reported the mean age of 35.5 years in their study of 146 cases. It is reported in the literature that it is more common in the female gender.^2^ In accordance with the literature, 53.4% of our cases were women.

Almost half of the cases with mediastinal neurogenic tumors are asymptomatic and are incidentally detected during routine chest radiographs. Clinical symptoms occur when the tumor reaches a large size or begins to compress on surrounding structures. The most commonly reported symptoms are cough, dyspnea, chest-back pain, and neurological abnormalities.^7,8^ In our study, 34.6% of the cases were asymptomatic. The cases most frequently applied to our clinic with complaints of back pain and chest pain.

Preoperative diagnosis of mediastinal neurogenic tumors is usually made by radiographic imaging. They are usually seen as a well-circumscribed lesion in the posteroanterior chest radiography. Contrast-enhanced thorax CT gives information about the location, size, density, contrast enhancement, calcification, and its relationship with neighboring structures. Thus, it is possible to predict the difficulties of resection. With preoperative CT, almost all cases can be diagnosed with neurogenic tumors radiologically.^8,9^ Approximately 10–20% of neurogenic tumors in the posterior mediastinum have a spinal canal component called dumbbell tumors.^5^ When the tumor is suspected to extend into the spinal canal, an MRI evaluation should be performed to detect the longitudinal extension of the tumor in the vertebra.^10^ The 18-FDG-PET-CT study is of no benefit in demonstrating whether neurogenic tumors are benign or malignant.^11^ Extension to the spinal canal was detected in only one case in our study.

Nerve sheath tumors are the most common in adults. Benign tumors in this group are schwannoma, melanotic schwannoma, and neurofibroma, while malignant tumors are malignant schwannoma or neurogenic sarcoma. Approximately, 98–99% of neurogenic tumors originating from the nerve sheath in the adult age group are benign.^5,9,10,12^ Benign lesions are seen in young and middle-aged adults. It is more common in women than in men. It usually occurs as a single lesion. In those with multiple, neurofibromatosis (Von Recklinghausen) disease is seen together.^2,3^ In our study, tumors of 19 (73.1%) cases originated from the nerve sheath, and 18 cases had schwannoma, and 1 case had neurofibroma. All of the cases were adult cases, and the tumor was benign in all the cases.

The most common mediastinal neurogenic tumors of childhood are tumors originating from autonomic ganglia. Ganglioneuroma, neuroblastoma, and ganglioneuroblastoma are included in this group. The average age of the cases is 2 years. About 25% of the cases are diagnosed under the age of 1 year, and 97% are seen before the age of 10 years.^4,9,12,13^ They arise from the sympathetic chain and ganglion cells in the adrenal medulla. Tumors that develop from fully immature undifferentiated neural crest cells are called neuroblastoma, ganglioneuroblastoma if they contain immature undifferentiated cells as well as mature ganglion cells, and ganglioneuroma if they contain fully differentiated ganglion cells. Spontaneous regression, ganglioneuroma, and ganglioneuroblastoma differentiation can be seen in neuroblasts. Ganglioneuromas constitute 10% of mediastinal neurogenic tumors. Ganglioneuroma is the most benign tumor of the neural crest.^12,13^ It can occur directly (primary) or as differentiation of neuroblastoma. Ganglionöroblastom is a form between ganglioneuroma and neuroblastoma, showing a histological structure in different differentiation. It is malignant but has a better prognosis than neuroblastoma. It is less common than neuroblastomas in total, but their incidence in the thorax is the same.^9,14^ In our study, 6 (23.1%) of the neurogenic tumors originated from autonomic ganglia. Ganglioneuroma was detected in 3 of our cases, and these 3 cases consisted of pediatric cases (3, 6, and 7 years, respectively). Ganglioneurobalastoma was detected in 2 cases, one of them was a child (2 years old) and one was an adult. Neuroblastoma was detected in a 1-year-old patient.

Patients with dumbbell tumors should be evaluated especially carefully. Extending into the spinal canal, tumor size and location make this type of tumor difficult to resect. It is very important to fully evaluate the presence and extent of extension to the spinal canal before surgery. In these cases, a multidisciplinary team of thoracic surgeons and neurosurgeons should cooperate to determine the most appropriate approach. The aim is to eliminate cord compression and prevent complications associated with the defect. In the same session, first a neurosurgical operation and then a thoracic procedure should be performed.^5,8,15^ To prevent injury to Adam Kiewickz artery, especially during dissection of the internal and posterior parts of tumors below the Th7 level, the origin and course of the artery should be seen with spinal angiography before surgery.^8^ In our study, the Dumbbell tumor was seen less frequently than in the literature. Angiography was performed in two patients to evaluate the Adam Kiewickz artery.

Some posterior neurogenic tumors located at the apex of the thorax extend to the cervical region. Tumors in this region always require special attention for surgeons due to their close relationship with important nerve and vascular structures and the difficulty of revealing the upper border of the tumor. Various approaches have been reported in these cases, such as a standard thoracotomy, thoracoscopy, lateral cervical approach, anterior thoracic approach, or a combination of these approaches.^5,15,16^

Thoracoscopy has been increasingly accepted as an effective and safe minimally invasive treatment modality for the treatment of posterior mediastinal neurogenic tumors as an alternative to thoracotomy. Posterior mediastinal located tumors are in an ideal location for the thoracoscopic approach. Better visualization of the mediastinum, reduction of surgical trauma, shorter hospital stay, less need for painkillers, and reduction in the risk of pulmonary complications are the most frequently emphasized advantages of the minimally invasive technique.^2,3,5,17^ The thoracoscopic approach provides a clear view of the tumor covered by the pleura. The tumor is usually encapsulated and can be easily mobilized from surrounding tissues. The use of endoclips is preferred for the neurovascular pedicles of the tumor.^8^ In our study, it was observed that the operation time and hospitalization were less in the thoracoscopy group.

It is reported that the most important factor affecting the thoracoscopy procedure is the diameter of the tumor. Tumor diameter greater than 6 cm, an extension to the spinal canal, localization of the tumor in the thoracic apex or costodiaphragmatic sulcus, and malignancy of the tumor are generally considered contraindications for the thoracoscopic approach. If the tumor is larger than 6 cm in diameter, the operative time, blood loss, conversion to thoracotomy, and complications increase significantly compared with small tumors.^2,4,15,16^ However, some studies are showing that even very large tumors can be resected thoracoscopically without any problem. Therefore, many authors consider the size of mediastinal neurogenic tumors not a contraindication criterion for thoracoscopic resection.^4,17^ A frozen section of the resection site can be performed to provide a tumor-negative surgical margin.^4^ In our study, the mean tumor size was 7.4 ± 1.9 cm in the thoracotomy group and 4.3 ± 1.9 cm in the VATS group.

Thoracoscopic resection in children is still controversial, as approximately one-third of all mediastinal tumors are neurogenic tumors and approximately 60% of them are malignant.^18^ Failure to obtain adequate tumor-negative surgical margins, tumor recurrence in the thoracic cavity, or at the tube thoracostomy site are the avoided complications.^19^ However, with the advances in surgical tools and techniques, complex thoracoscopic procedures have begun to be used more successfully in the pediatric group.^4^ In adults, VATS is usually the preferred method for surgical resection, as almost 95% of cases are benign.^4,17^

Rarely conversion to thoracotomy has been reported due to technical difficulties, operative complications, or suspected malignancy.^8^ Liu et al.^20^ reported that conversion to thoracotomy is not necessary in cases where the tumor is smaller than 8 cm. The frequency of conversion to thoracotomy due to difficulties such as bleeding or pleural adhesions is between 6% and 22% in the literature.^3^ In our study, one patient needed conversion to thoracotomy.

The most common complication after thoracoscopic removal of the mediastinal neurogenic tumor is Horner’s syndrome.^21,22^ The occurrence of Horner’s syndrome in the postoperative period is related to the localization of the tumors in the upper thoracic region rather than the type of surgical resection.^4^ Yang et al.^23^ reported a higher incidence of brachial plexus injury with thoracoscopic resection. Also, failure to detect the extension of the tumor to the spinal foramen or canal may cause inadequate resection, nerve damage, or intratumoral bleeding.^5^

Consequently, most of the posterior mediastinal neurogenic tumors are benign. Surgical resection of these tumors has excellent results. The surgical approach should be decided according to the size and location of the tumor and its extension into the spinal canal. The majority of benign tumors without intraspinal involvement can be completely resected thoracoscopically. With a thoracoscopic procedure, the duration of hospital stay and the level of pain can be reduced and a better cosmetic result can be achieved. Thoracotomy is an appropriate surgical approach for large-sized and malignant tumors.

**Main Points**

- Schwannomas are the most common type of neurogenic tumors.
- Generally, neurogenic tumors detected in adults are benign, while childhood neurogenic tumors are malignant.
- Thoracoscopy is an advantageous method that can be applied safely in appropriate cases in the treatment of neurogenic tumors.
- Patients with dumbbell tumors should be evaluated by a multidisciplinary team of thoracic surgeons and neurosurgeons.

## Figures and Tables

**Table 1.** Comparison of Thoracotomy and VATS Cases

	Thoracotomy (n = 14)	VATS (n = 12)	P
Age	34.2 ± 22.3	45.4 ± 21.6	.207^a^
Gender (male/famale)	5/9	7/5	.448^b^
Tumor size (cm)	7.4 ± 1.9	4.3 ± 1.9	.001^c^
Histopathological type			.318^d^
Schwannoma	9	9	
Neurofibroma	3	1	
Ganglioneuroma	1		
Neuroblastoma	1	1	
Ganglioneuroblastoma		1	
Paraganglioma			
Surgery duration (min)	101.7 ± 27.8	77.92 ± 24.35	.014^c^
Postoperative stay (day)	7.4 ± 4.0	4.7 ± 1.7	.040^a^
Recurrence (yes/no)	2/12	0/12	.483^e^
Death within 30 days	–	–	N/A

All values are given as mean ± standard deviation or number, N/A: Not Applicable

a Independent One-Sample *t*-test

b Yate’s continuity correction

c Mann-Whitney *U* test

d Chi-Square test

e Fisher’s Exact test
